# Locked in grief: a qualitative study of grief among family members of missing persons in southern Sri Lanka

**DOI:** 10.1186/s40359-021-00675-7

**Published:** 2021-10-29

**Authors:** Amila Isuru, Padmakumara Bandumithra, S. S. Williams

**Affiliations:** 1 Department of Psychiatry, Faculty of Medicine and Allied Sciences, Rajarata University of Sri Lanka, Anuradhapura, Sri Lanka; 2 District General Hospital, Hambantota, Sri Lanka; 3 Department of Psychiatry, Faculty of Medicine, University of Kelaniya, Ragama, Sri Lanka

**Keywords:** Grief, Disappearances, Psychosocial needs, Missing individuals

## Abstract

**Introduction:**

The psychological and social issues experienced by family members of missing persons are different from normal grief following the death of a loved one. The term “Ambiguous loss” describes this psychological phenomenon. Ambiguous loss acts as a barrier to adjusting to grief, leading to symptoms of depression and intra and interpersonal relational conflicts. An in-depth phenomenological understanding of this subjective experience is important.

**Method:**

A qualitative study was conducted among close family members of persons who had gone missing during the civil conflict and the 2004 tsunami in southern Sri Lanka following formal ethical approval from an university ethics review committee. Purposive and snowballing sampling methods were used to recruit the participants. Theoretical sample saturation was achieved with 24 family members of missing persons. Responders were mothers, fathers, wives, husbands, and siblings of missing individuals. In-depth interviews were recorded with the help of a semi-structured guide, after informed consent. The recordings were transcribed and coded by three independent investigators. The investigators through consensus arrived at the phenomenological themes and grounded them through reflexivity. The triangulation process involved cross-checking observational notes made by the interviewers and consulting the interviewees.

**Results:**

We interviewed 24 first degree relatives of missing individuals. Twenty-one of the interviewees were unsure about the fate of the missing individual, while three of them believed the missing individual to be dead. Of the 24 missing individuals, 20 were males and 18 had gone missing in civil conflicts and 6 in the Indian Ocean Tsunami. Six predominant phenomenological themes were identified. Those were lack of closure, hope, guilt, helplessness, perpetual suffering, and an emotional vacuum. These phenomenological experiences are highlighted by the interviewees through a range of utterances that hold profound cultural, social and emotional significance of unresolved and vacillating grief.

**Conclusion:**

The highlighted phenomenology of grief in surviving family members of those who go missing following traumatic events demands a response from health and social services in every country that experiences disaster. The surviving loved one is ‘locked in grief’ indefinitely and future research on evidence-based interventions to overcome this predicament is warranted.

## Introduction

Sri Lankan has experienced natural and man-made disasters over the last five decades, which have resulted in many persons being classified as missing and not accounted for. Family members lost their loved ones in the context of an ethnic conflict which spanned over three decades and ended in 2009, numerous youth uprisings between 1971–1989, and the Indian Ocean Tsunami in 2004 [1].

The UNHCR documents more than 34,000 complains by family members of persons who have gone missing during the civil conflicts. Of them 5100 was of serviceman who were classified as missing during combat [2].

The Indian Ocean tsunami in 2004 affected Sri Lanka in unprecedented ways and the Centre for National operations Sri Lanka, reports 31,187 deaths. A further 4280 were documented as missing as their remains were not found [3].

People go missing everyday amidst civil conflicts, violence and natural disasters across the globe. There are thousands of Americans grieving over missing individuals after the September 11, 2011 attack [4]. Other countries like Bosnia and Herzegovina, Kosovo and Nepal too report a high number of civilians as missing in civil conflicts [4].

When someone goes missing and unaccounted for, the family members left behind are on a perpetual mission to clarify the fate of the missing individual [5]. They contemplate all the possibilities, but the finality of the death of a loved one is elusive and they constantly vacillate between hope and despair [5, 6]. The grief process is not initiated since there is no dead body. This unique suffering experienced by family members of missing individuals is described by the term Ambiguous loss [5]. The loss of a loved one is in itself, difficult. When ambiguity is added to it, the results are agonizing and immobilizing [5]. Furthermore, ‘ambiguous loss’ is compounded by legal, financial and social ramifications [7, 8]. Therefore, ambiguous loss is a different kind of loss from a loss by bereavement [9]. In depth understanding of the lived personal experience of close family members is necessary to support and meet the needs of these persons who are unable to achieve closure.

A broader phenomenological understanding of the psychological and social effects on the close family members of those classified as ‘missing’ is necessary, to address the needs of these affected individuals. Resolving the problems in families of missing individuals is an important facet of any post war reconciliation process as issues related to missing persons act as reminders of past conflict [7].

The aim of our study was to understand the nature of grief through qualitative means in family members whose loved ones disappeared in the context of civil conflicts between 1987–2009 and the Indian Ocean Tsunami of 2004 in southern Sri Lanka.

## Methodology

### Conceptual framework

The emotional experience following a person going missing is different from the grief following the death of a loved one [4]. As there is no confirmation of death and the mortal remains are not received, the natural process of grief is not initiated [10]. The missing person is therefore physically absent but psychologically present in the minds of the loved ones.

We aimed to describe the phenomenological experience of grief and understand the emotional and social needs of family members of missing persons. A good understanding of their lived experience and needs enables health care professionals to plan an effective intervention. Previous qualitative studies on this are mostly from high income countries [11]. Therefore, it is important to understand the experience of family members of missing persons in the south Asian context, as it may be different considering the diverse cultural and socioeconomic factors.

### Study design and sampling

Family members of missing individuals from Galle, Matara and Hambantota districts were identified with the help of the Grama Niladhari (local administrative officer). First degree relatives of missing persons were interviewed after informed written consent. Purposive sampling was used due to the sensitive nature of the research topic. The sampling tried to achieve maximum variation in terms of age, relationship to the missing individual, gender, social class, context in which the missing took place, ethnicity and religion.

Southern province is the seventh largest and 3rd most populated province in Sri Lanka. The province consists of three districts which home nearly 2.5 million persons. The main income of the population is from farming and fishing.

### Data collection

We interviewed 24 persons between January and September 2015 to achieve data saturation. The qualitative research methodology included semi-structured and in-depth face to face interviews which were tape recorded.

Semi-structured interviews focused on socio-demographic factors of the missing individual and interviewee—the situations in which disappearance had occurred, their belief in the fate of the missing individual and psychosocial needs of the interviewee.

The interviews were conducted in a safe space mostly in the home of the interviewee in a sensitive manner. The interviewee was given permission to stop the interview at any point if they found the interview to be emotionally overwhelming. Initially, the interview started with general questions and then moved on to more specific information.

Data collection was conducted by two medical doctors who had post graduate qualifications in Psychiatry. The respondents were clearly informed that participation in this research did not entail any financial, legal or other aid. It was also made clear that it was not a diagnostic interview with a view to providing medical or psychological therapy. The researchers had prior training to avoid a clinical interview pattern that could potentially introduce clinically driven misconceptions. Interviews were conducted in a place chosen by the interviewee to minimize the effect of an unfamiliar backdrop.

Interviews were conducted in Sinhala language, which is the main language spoken by people in southern Sri Lanka. Each interview lasted approximately 1 h. The researchers did not ask direct questions and facilitated the flow of information with open ended and probing questions. At the end of the interview, the interviewees were given an opportunity to express any additional concerns or thoughts. The collected data was stored confidentially by the researchers with the interviewee being masked to avoid any identification.

For methodological rigor, the following steps including prolonged engagement with interviewees, detailed descriptions from participants, negative case analysis, co-coding of transcripts and use of an audit trail were employed. Triangulation was achieved by comparing the transcript with observational notes done by the interviewers. Further the interviewers who were involved in data analysis examined their own values, beliefs and their position on this sensitive issue of missing individuals, to minimize bias in the analysis.

### Data analysis

The anonymized audio recorded interviews were transcribed. A reflective approach to analysis was taken. Each transcript was analyzed by two researchers. Manual coding was done instead of computer program-based coding due to non-availability of qualitative analytic software. A qualitative content analysis method was used, and transcripts were read through multiple times with codes generated by grouping relevant phrases and words together. Grounding was done by line-by-line analysis and constant comparison by two researchers.

Information from each section was compared and grouped until similar phenomenology were identified. Constant comparison of codes was performed. The final frame was arrived at by consensus.

### Ethical approval

Ethical approval for the study was obtained from the ethical review committee of the Faculty of Medicine, University of Kelaniya.

## Results

The majority of interviewees were mothers [12] followed by fathers [4]. The age range was 44 years to 81 years. Disappearances had taken place between 1988 and 2004. None of the interviewees had a history of mental disorder. The characteristics of the missing persons are listed in Table 1. The characteristics of those interviewed are listed in Table 2.

**Table 1:** Demographic characteristics of missing individuals

	Number of individuals
1. Gender	
Male	20
Female	4
2. Civil status	
Married	8
Unmarried	16
3. Occupation	
No occupation	11
Non skilled	1
Skilled	8
Professional	4
4. Level of education	
No formal education	2
Up to grade 5	2
Grade 6 to GCE O/L	9
Advanced level	6
Degree	5
5. Ethnicity of disappeared persons	
Sinhala	21
Tamil	0
Muslim	3
6. Religion of missing person	
Buddhism	21
Catholicism	0
Hinduism	0
Islam	3
7. Context in which person went missing	
Tsunami	6
Civil conflict	18
8. Breadwinner of the family	9

**Table 2:** Socio-demographic characteristics of interviewees

	Number of individuals
1. Gender	
Male	6
Female	18
2. Civil status	
Married	17
Divorced	1
Widowed	6
3. Relationship to the missing person	
Mother	13
Father	4
Wife	3
Husband	1
Brother	1
Sister	2
Son	0
Daughter	0
4. Education	
No formal education	7
Up to grade 5	8
Up to O/L	4
Up to A/L	3
Degree	2
5. Occupation	
Unemployed	17
Unskilled	1
Skilled	2
Professional	4
6. Ethnicity	
Sinhalese	21
Muslim	3
7. Religion	
Buddhist	21
Islam	3
8. Belief on the status of the missing person	
Firmly believed as dead	3
Firmly believed as living	0
Not sure	21

The following phenomenological themes emerged in the interviews: Lack of closure, hope, guilt, helplessness, perpetual suffering and an emotional vacuum (Table 3).

**Table 3:** Identified themes and codes

Theme	Code
Lack of closure	Not giving in to grief Ambiguous nature of the loss of the loved one
Hope	Hope was a way of maintaining connectedness to the missing person Giving up on hope implied the acceptance that the missing person would not return alive Helped deal with the pain
Guilt	It was wrongful to be joyful and enjoy life
Helplessness	Lack of reliable information about the missing person despite trying for many years Financial and bureaucratic barriers to seeking justice Financial hardships secondary to losing breadwinner of the family and spending large sums of money to search for the missing loved one
Perpetual suffering	Experiencing intense psychological distress including prolonged grief, depression and suicidal ideation for decades
Emotional vacuum	Families in particular expressed unfulfilled grief

### Lack of closure

The nature of the grief is complex. It vacillates between searching for the missing, while also having given up.

A mother said, “If my son is alive, he will somehow come to see me. Even if he was tightened up with 7 iron chains, he will find the way home. Now it is more than 25 years since he became unaccounted for…. So, I don’t think he is alive. We are giving alms annually in remembrance of him. But we have not given up searching for him…I went to a fortune teller last week also to find out if my son is alive somewhere”.

The lack of closure and ambiguity is further highlighted by the statement of this sister of the loved one. “It is nearly 27 years since my brother disappeared. We do religious activities and bless him. But we did not perform death rituals or give Panshukuula (A Buddhist blessing performed after death). It is bad if we do such things to someone alive. Our uncles and villagers asked us to give alms to Buddhist priests and invoke merits. My parents and I don’t want to do that since we believe that he is alive somewhere.” There is a reluctance to give in to complete grief.

### Holding on to hope

Hope is pivotal to the experience and fuels the ceaseless emotional search to reconnect with the missing loved one. The following statement by the sister of an abducted male, shows the yearnings of a mother. “My brother was abducted by men in military uniform in 1989. He had just completed his ordinary level exam with eight distinctions. My mother was continuously in a mission to find him… it was her daily routine until her death. She has never allowed us to close the front door of our house, believing that my brother would return home.” Connectedness to the missing person can be enhanced by hope, which in turn may assist the complicated bereavement reaction [13].

Hope gives a reason to the survivor to continue living. A father of an army soldier said, “Believing that my son will return, helps to keep me going”. It also helps mitigate the pain. A father said, “I have a belief and feeling that whatever people said, my son is alive. Actually speaking, I did not experience an unbearable shock since I believe that my son is alive somewhere. The idea in my mind is that I need to search for my son more and more”.

Hope is enhanced by clutching to selective reinforcers. “I think my son is alive. I have been searching for him for more than 25 years. We listen to fortune tellers and do all sorts of sorcery. Fortune tellers say that my son is alive. A fortune teller said that he would definitely return home. Then we went to a place where they do some sorcery called “Anjanam Eli”. That person also confirmed that my son is alive and is in good health. He further said he could clearly see him alive”.

### Guilt

The guilt precluded normal life and the ability to move forward. A mother said, “I haven’t cooked milk rice (a traditional meal) or participated in any festive event so far. How can I be happy without my son?”. In Sri Lankan culture guilt is not expressed explicitly but in more indirect and metaphorical ways [12]. Therefore, the guilt of this mother held her back from participating in the traditional festivities that are a part and parcel of meaningful enjoyment for south Asians.

### Helplessness

They faced numerous hardships. A wife said, “My children and I were left with no income after the disappearance of my husband. My husband planted jack trees in our garden. We ate jack fruit when there was nothing to eat. I do not hesitate to say this publicly. We ate plain bread and drank water. My sister helped me from time to time. She gave us rice whenever she could”.

The financial resources were further depleted in the quest for the missing person. “I lost my son 25 years ago. We lost all our sources of income, as we spent time looking for evidence of my missing son. We sold all our properties one by one. Now we don’t have any. We are now struggling to meet our day-to-day expenses”.

The efforts at seeking justice were fruitless. A wife said, “I went to make a complaint. They chased me away. We could not make a complaint. But I took courage and went again with a neighbor. They chased us away again. So, I did not to seek justice. On the other hand, we did not have money to file a court case either”.

### Perpetual suffering

The emotional distress is relentless. A mother said, “Since the day my son disappeared, I haven’t slept. I wake in the middle of night and cry. The other children asked me to forget about it. How can I do that? He is the world to me. I am just living. If someone gives me food, I eat”.

It results in somatic ailments and health seeking behavior. Another said, “It was very difficult to bear my son’s loss. I was unwell for a long period of time and suffered a lot. I had various difficulties, pains and aches. I had to take a lot of medications and saw several doctors. At the end, the doctor said that I don’t have an illness. Then the doctor had inquired from my daughter whether I have any worries. Then she told him that I am worried about the disappearance of my son who was serving in the army. Then Doctor asked me to go to my village, socialize with my relatives, get involved in religious activities and listen to the radio. He also explained that I am not the only mother whose son was unaccounted for”.

Others had prolonged grief and depression. A mother said, “The pain of losing my son was so overwhelming that I went out of the house at midnight when all the family members were fast asleep, to jump into the well and kill myself. My husband had seen me going towards the well and shouted. Then all the family members woke up and took me back to the house. No one can understand the pain I am going through. I know everyone dies, but my son died untimely, and it was very unfortunate to lose him. I feel very lonely without him. It would be better if I die. Then again, I think I have two other children. I do some meritorious act every day for him”.

### Emotional vacuum

The females in particular expressed an unfulfilled emotional need. A mother said, “If my son had been with me, things would have been much better for me. He would have taken me to the doctor, and I am sure he would have been there for me in my difficulties”.

### The needs of family members of missing persons

In the semi structured interview, the pressing need in eleven respondents was to know the fate of the missing individual. Even then, whether such knowledge would console the living is questionable. As one mother summed it up, “I just want my son. I don’t want anything else”.

Others identified financial needs and the need for psychological support. Of the 24 interviewees, 13 felt they were stigmatized and marginalized by the rest of the community.

## Discussion

This study further illustrates the nature of the lack of closure and ambiguity experienced by family members of missing individuals even after two decades of the disappearance of their loved one. Ambiguous loss disenfranchises the normal grief process and leads to a prolonged ‘vacillating grief’. A grief which vacillates between hope and despair. The lack of a closure, mitigating any efforts to move on through the interplay of guilt and the denial of the inevitable. An intense helplessness and perpetual suffering that struggles to make sense of the predicament and to attain cognitive consonance in the midst of uncertainty. The phenomenon captured by the term “living in limbo” [14]. Lack of closure is fundamental and a recent study from Sri Lanka showed that even subsequent confirmation of death prevented psychological morbidity in the form of depressive disorder and prolonged grief [10]. In this study, among 20 out of the 24 interviewed, no religious or farewell ceremonies had been performed for the missing person. Such ceremonies undoubtedly facilitate closure [15, 16]. Family members of missing individuals in our study, often neither accept death certificates nor accept reparations. Such acceptance would be contrary to their hope due to the lack of closure as shown in a similar study in Nepal [17].

In Sri Lankan culture, people turn to fortune tellers to know the possible outcome during uncertainty. The false information provided by fortune tellers, renews hope. This further confounds the situation and leads to a renewed effort to seek out the missing person spending large sums of money by selling their property or borrowing. We identified one other study that reported this kind of culturally sanctioned behaviour of family members of missing individuals [17].

Bowlby described that failed attempts to restore missing relationships often results in perpetual distress and agony [18]. This perpetual suffering was evident in our findings. It had psychological and psychosomatic ramifications. The higher risk of developing physical and mental illness in the context of chronic stress is well documented [9].

Studies on psychological consequences of family members of missing persons are limited [9]. A study done in Bosnia and Herzegovina reported that symptoms of post-traumatic stress disorder (PTSD) was significantly higher in wives of missing individuals than in those who had been exposed to other traumatic experiences during the political unrest in their country [19]. Most of the research looking at psychological morbidity has focused on PTSD. But ambiguous loss is essentially a relational issue, and the trauma is ongoing.

A study done by ICRC in Sri Lanka reported that family members of missing individuals experience a high level of psychological morbidity. Of 321 participants, 51% showed anxiety symptoms, 58% showed symptoms of depression and 15% showed symptoms of PTSD [20]. Professor Pauline Boss showed that people who experience ambiguous loss are at a higher risk of developing depression, anxiety disorders, and substance use disorders [5, 21]. However, these studies focused on symptoms of mental illnesses and categorical diagnoses was not made.

Caution should be exercised when making a diagnosis in family members of missing persons. When they are screened for depressive disorder, prolonged grief disorder or PTSD, it should be kept in mind that symptoms of ambiguous loss can be attributed to mental disorders. On the other hand, most symptoms of depression, prolonged grief disorder and PTSD tend to overlap [22].

Survivor guilt impedes on enjoying life as before. Family members left behind believe that they give up on the missing person if they started living normally. In our study, family members of missing individuals did not attend festive events such as weddings and did not cook special food items that they would usually prepare for festive events. Such actions created self-imposed barriers to their happiness.

The complexities are many as highlighted in previous studies. There is a gendered impact on disappearances [23]. When the disappearances occur in the context of civil conflicts the majority of disappeared persons are males [23]. Therefore, the economic, social and psychological impact on female family members is relatively high [24, 25].

Disappearances cause greater impact on older females [23]. In most cultures, children, especially male children, support their parents in their senior ages. When their children become unaccounted for, they are deprived of security and financial support which could have been received if their missing son had lived with them [23].

Wives face the dual challenge of managing the family responsibilities in the absence of the missing husband, and the grief stemming from the loss [23]. There can be many bureaucratic hurdles in accessing the husband’s salary, bank account, and other properties in the absence of a death certificate [23]. In some cultures, bodily markings are used to signify the status of marriage. In Tamil culture in Sri Lanka, married women are expected to wear ‘thali’ (a jewellery item) and kungumappottu (a red mark on the forehead) [23]. When a widow or a wife of a disappeared person wears such bodily markings, the symbols are considered as an ill omen. As a result, wives of missing persons do not enjoy the same privileges as their married counterparts, which may discourage them from attending public events [23].

Families in southern Sri Lanka live with their extended family which can be helpful in many ways in the face of adversities. At the same time, living with an extended family can be a great source of stress as well. The majority of interviewees in our study identified that their family members are helpful when coping with the stress of ambiguous loss. However, some mothers in this study whose sons went missing reported that family members blame them and remove the photograph of the missing son as they are always mourning looking at the son’s photograph.

When a family member is physically absent, but psychologically present most of the time, the family members’ perception of who is inside or outside the family system is blurred [5]. Ambiguous loss confuses boundaries of family members resulting in boundary ambiguity. Most of the missing individuals are young males in southern Sri Lanka [9]. In Sri Lankan culture, male partners ensure the protection of the family. In their absence, families feel that they are not safe as before [20]. In the absence of the breadwinner of the family, others have to take the responsibility of earning money which can be quite challenging. Most of the missing individuals in our study sample were males and some of them were breadwinners of the family. Some of the family members in our study also described how insecure they were in the absence of the missing person. Hence ambiguous loss disturbs relationships and dynamics of everyday life in the family.

### Limitations

It should be noted that there may have been other losses and stressful events which may have cumulatively affected the grieving process. The sampling is from a specific geographical location and socio-cultural context.

There is also a possibility that the researcher could have been perceived as someone who could deliver assistance or compensation to the interviewees. This could lead to exaggeration of the impact of the disappearance. Some families were involved in socio-political activities to determine the truth or seek justice with regard to the missing individual.

## Conclusions

Family members of missing individuals experience a unique kind of grief, vastly different from grief following bereavement. Lack of closure, holding on to hope, guilt, helplessness, perpetual suffering and an emotional vacuum impact the loved ones of the missing. There is a vacillation between hope and despair that prevents acceptance of the loss. The individuals are essentially “locked-in grief”, unable to move on.

This study illustrates the unique phenomenology of grief experienced by family members of missing persons and its disabling nature. Further research on evidence-based interventions to mitigate this suffering is imperative.

## Data Availability

The data sets used and/or analysed during the current study are available from the corresponding author on reasonable request.
